# Biomechanics of Central Incisor Endocrowns with Different Lengths and Milled Materials after Static and Vertical Loading: A Finite Element Study

**DOI:** 10.1155/2024/4670728

**Published:** 2024-03-31

**Authors:** Waleed M. S. Alqahtani, Salah A. Yousief, Raafat Tammam, Rami M. Galal, Ali Brakat, Hend Mohamed El Sayed, Ala’a Kamal, Mohammed Noushad, Mohammad Zakaria Nassani

**Affiliations:** ^1^ Department of Prosthetic Dentistry College of Dentistry King Khalid University Abha, Saudi Arabia, kku.edu.sa; ^2^ Department of Restorative and Prosthetic Dental Sciences College of Dentistry Dar Al Uloom University Riyadh 13313, Saudi Arabia, dau.edu.sa; ^3^ Department of Crown and Bridge Faculty of Oral and Dental Medicine Al Azhar University Assuit Branch Cairo 71524, Egypt, alazhar-gaza.edu; ^4^ Department of Prosthodontics Faculty of Dentistry Assiut University Assiut 71515, Egypt, aun.edu.eg; ^5^ Department of Fixed and Removable Prosthodontics National Research Centre Cairo, Egypt, nrc.sci.eg; ^6^ Department of Restorative and Prosthetic Dental Sciences College of Dentistry Dar Al Uloom University Riyadh 13313, Saudi Arabia, dau.edu.sa; ^7^ Restorative Dentistry Conservative Dentistry Department Faculty of Dentistry Cairo University 11 EL-Saraya Street Manial Cairo 11553, Egypt, cu.edu.eg

## Abstract

**Statement of Problem:**

The performance of central incisor endocrowns with varying crown heights and different computer‐aided designs and computer‐aided manufacturing materials is not clear.

**Purpose:**

The aim of this study was to compare and assess the stress distribution and failure possibility of endodontically treated central incisor protected with endocrowns with different heights, with various CAD–CAM blocks such as IPS e.max CAD, Katana Zirconia, and Zolid Fx Zirconia.

**Materials and Methods:**

A root canal‐treated central incisor (plastic model) restored with an endocrown was scanned with a laser scanner to prepare a control model with a CAD software and then transferred to an FEA software. Proposed crown heights were 2, 4, and 6 mm. The model that was duplicated and restored with CAD–CAM blocks, IPS e.max CAD, Katana Zirconia, and Zolid Fx Zirconia were tested as endocrown materials. Bone geometry was simplified to be two coaxial cylinders in all models. Stress distributions under 50 N axial and oblique (with 135° angle from the vertical plane) loading were analyzed. Each model was then subjected to two occlusal loading conditions—the lingual slope of the incisal edge and the junction between incisal and middle thirds. Eighteen runs and calculations were performed to determine the endocrown height and material effect.

**Results:**

The results showed a minor or negligible effect of changing the endocrown material. Increasing endocrown height was shown to reduce stresses and deformations on most of the model components (bone, gutta‐percha, periodontal ligament, and endocrown), except root and cement. Differences in deformations and stresses between the two models of 4 and 6 mm were relatively smaller (ranged between 1% and 30%) compared to those between the 2 and 4 mm models (ranged between 10% and 400%).

**Conclusions:**

The material used to fabricate endocrowns did not show considerable effect on the underlying structures. However, the endocrown design (2, 4, and 6 mm height) was shown to affect all components of the studied systems. Increasing endocrown height is recommended for bone, periodontal ligaments, and endocrown body, as it reduces stresses and deformations. On the other hand, it dramatically increases stresses on the root and cement layer. Smaller endocrown sizes represent an acceptable treatment option when there is a healthy periodontal state, while using larger sizes will be more suitable when there is a periodontal compromise with bone loss.

## 1. Introduction

According to the existing literature, endocrowns may perform similar to or even surpassing conventional treatments such as traditional posts, direct composite cores, or inlays/onlays [1].

Clinical trials indicate that endocrowns have a success rate ranging from 94% to 100% [2]. For example, endocrowns exhibited stronger fracture strength than traditional restorations in both posterior and anterior teeth, according to global research. However, when they were compared to conventional restorations, exclusively in the posteriors (subgroup analyses), insignificant differences had been observed [1, 3–5].

Endocrowns are used to restore teeth having clinical crowns with minute height and calcified, short, or curved roots, making traditional posts unfeasible [6]. Indicated also with patients having small interocclusal distances, preventing the ceramic veneer and metal or ceramic framework from being thick enough [7].

Endocrowns have been shown to be more resistant to fracture than conventional crowns when cast posts are used or a fiber post and resin core [8, 9]. They are extremely simple to use and take less time in the clinic. Fast preparation, minimum chair time, being relatively cheap, easy handling, and good esthetics are advantages of endocrowns [10].

Because of the limited tooth surface area available for adhesion, and the greater crown height of premolars and anteriors, endocrowns in these teeth have had less success than in molars [11]. Therefore, endocrowns should only be used on posterior teeth [12]. During physiological loading, although leucite ceramic endocrowns in incisors may fracture, lithium disilicate ceramic endocrowns have been shown to be resistant to failure. For anterior dental restorations, posts, and prosthetic crowns are still the restorations of choice [13].

Endocrowns can be manufactured from a variety of materials, such as feldspathic, lithium disilicate‐reinforced ceramics, hybrid resins, and new CAD/CAM ceramics and resins. Regarding the literature in the studies, mechanical characteristics and fractures in endocrowns have been tested with mostly mechanical methods and finite element analyses [14–16].

Ceramic endocrowns may be advised for anterior teeth replacement due to these benefits, as well as their esthetic appeal. Since the crowns of incisors have more height (10.5 mm) and narrower (7.0 mm) compared to molars (7.5 mm height, 10.0 mm diameter at the cervical line zone), the biomechanics of incisors differ from those of molars. According to the equilibrium of the lever, bending movements of restorations with incisors are more than on molars. Furthermore, the adhesion area with endocrown with incisors is about 30 mm^2^, which is two times smaller than that of molars (60 mm^2^), thereby reducing the retention of these restorations [13].

The Ferrule concept is very important in adding to the properties of the tooth, foundation, and dowel the capability of resisting loads and decreasing the fracturing possibility by decreasing force concentration at critical sites.

In dental biomechanical research, the FEA has been widely utilized to assess the stress distribution on materials and/or oral tissues [2]. The aim of our study was to use 3D FEA to examine the strength of incisor teeth repaired with ceramic endocrowns of various heights, and CAD–CAM materials. The null hypothesis assumes that incisor restorations with different materials result in no significant differences. In addition, the different endocrown heights have no significant difference. The limitation of this study was that it did not include any type of margin preparation.

## 2. Materials and Methods

**Figure 1:**
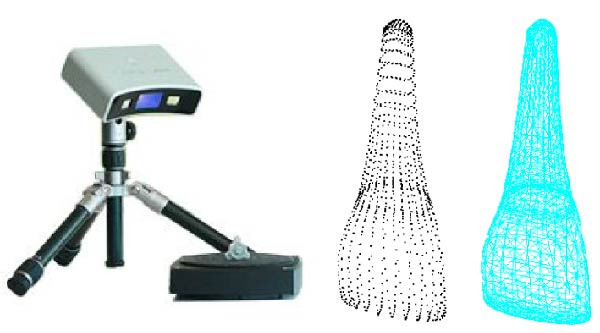
Laser scanner and scanned tooth as cloud of points and after creating its surface.

A 3D model for a central incisor was obtained by 3D scanning of a sample plastic tooth. The tooth geometry was acquired by using a laser scanner (Geomagic Capture, 3D Systems, Cary, NC, USA). Such scanner‐produced data file containing a cloud of points coordinates, see Figure 1. An intermediate software was required (Rhino 3.0‐McNeel Inc., Seattle, WA, USA) to trim a newly created surface by the acquired points. Then, the solid (closed) tooth geometry was exported to the finite element program in STEP file format. The model restored with a shoulder finish line endocrown has 3° taper, with cut levels (heights) of 2, 4, and 6 mm, which were used to create three models (or cases) for comparing endocrown heights, in addition to the material used. Those three materials for endocrown were tested as listed in Table 1, where all the materials used in this study were assumed homogeneous, isotopic, and linearly elastic.

**Table 1:** Material properties [17].

Material	Modulus of elasticity (MPa)	Poisson’s ratio
Cortical bone	13,700	0.30
Spongy bone	1,370	0.30
Periodontal ligament (PDL)	0.0689	0.45
Root (dentine)	18,600	0.30
Enamel	84,100	0.33
Gutta‐percha	14,000	0.40
Cement (resin of 30 *μ*m)	8,300	0.35
Crown 1: IPS e.max CAD	97,500	0.24
Crown 2: Katana Zirconia	205,000	0.25
Crown 3: Solid Fx Zirconia	200,000	0.31

Cortical and spongy bone were simplified as geometry to be two co‐axial cylinders with 16 and 14 mm diameter, 24 and 22 height, respectively. A set of Boolean operations was performed to create PDL, 30 *µ*m thickness cement layer, root cavity in bone, canal, etc., to create the final three models.

The meshing of the three models’ components was performed by using the 3D brick solid element “187,” which has three degrees of freedom (translation in main axes directions). The resulted numbers of nodes and elements are listed in Table 2, and samples of the model components and its mesh are presented as screenshots from ANSYS in Figure 2.

Figure 2Sample models’ components and its meshing: (a) cortical bone, (b) cortical and spongy, (c) meshed PDL, (d) remaining tooth in Models 1 and 2, (e) cement layer in Models 1 and 2, (f) endocrowns in the three models, and (g) final meshed models.

**Figure:**
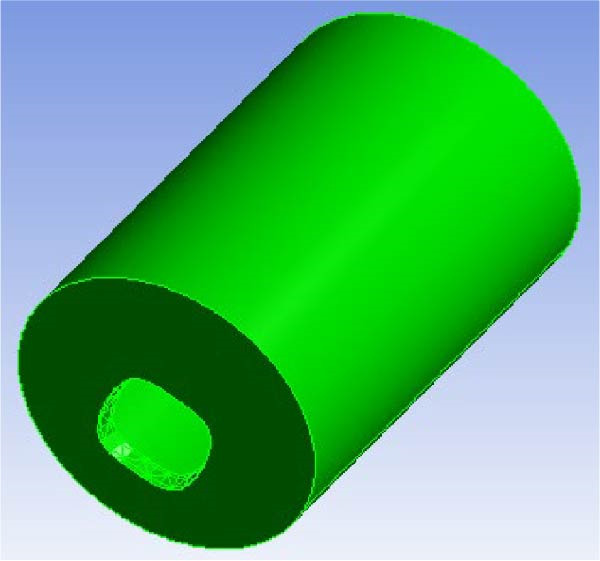
(a)

**Figure:**
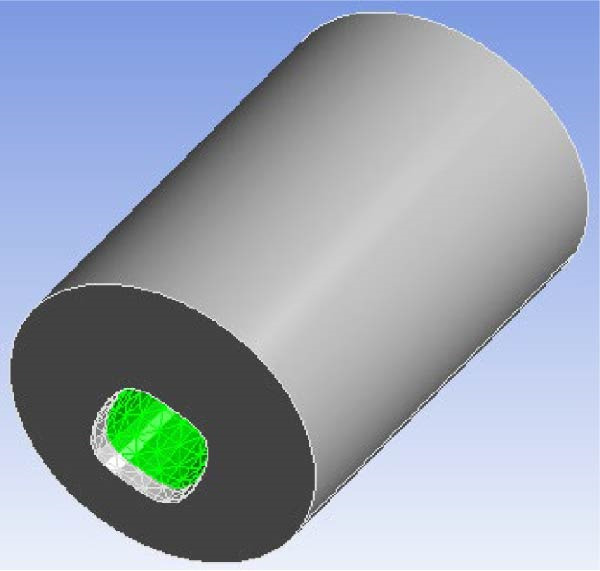
(b)

**Figure:**
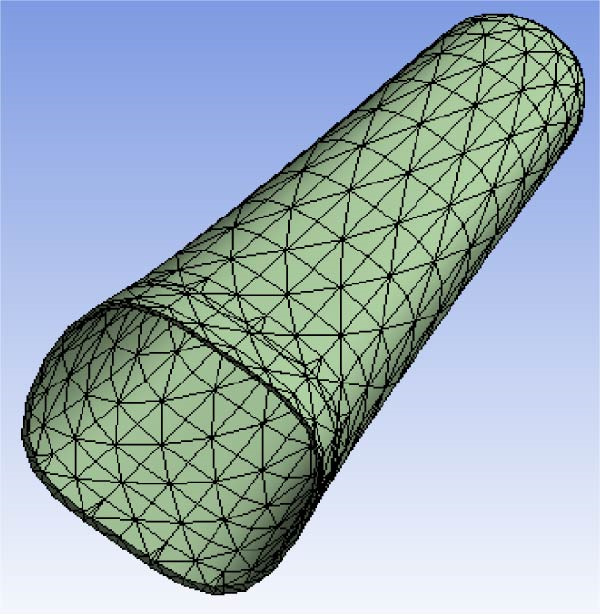
(c)

**Figure:**
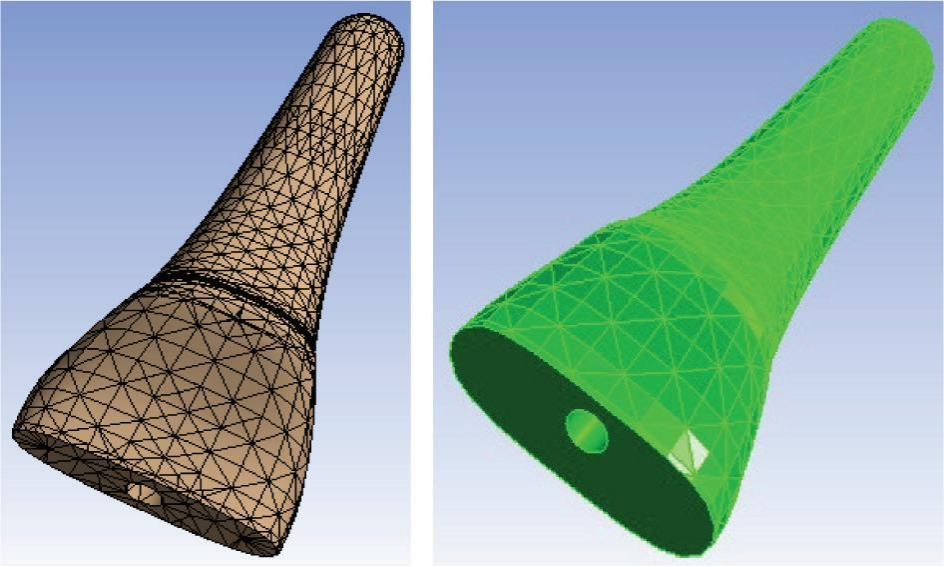
(d)

**Figure:**
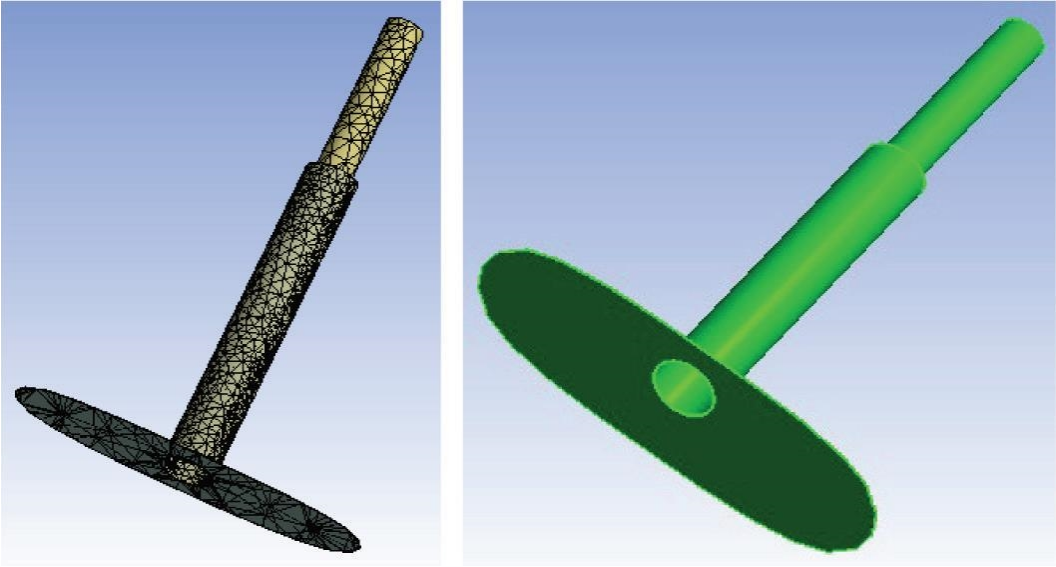
(e)

**Figure:**
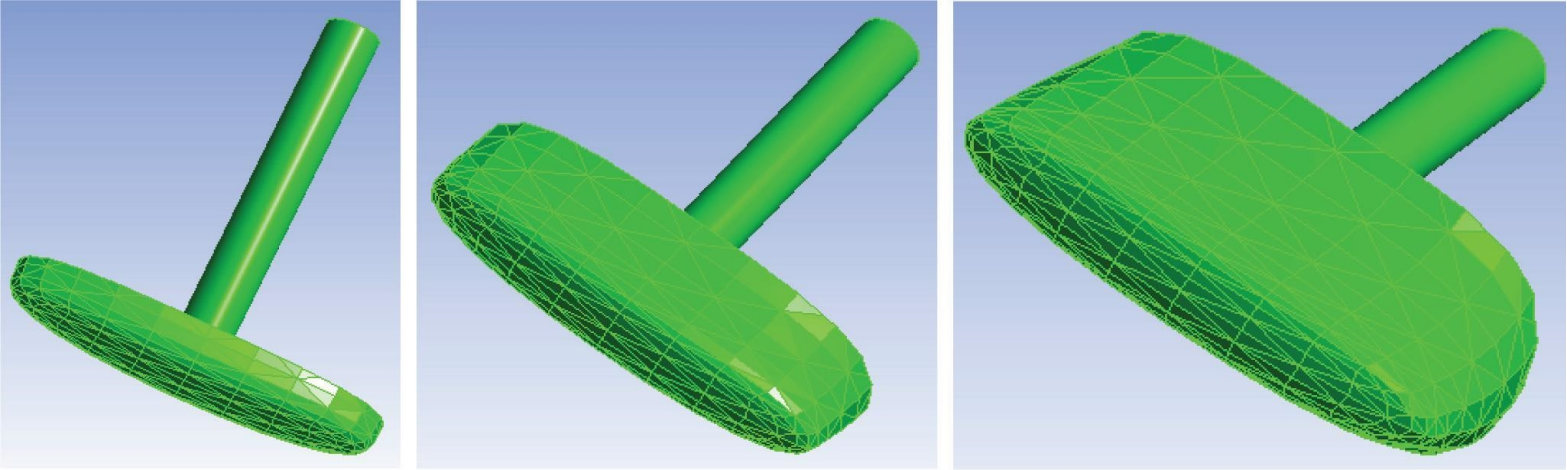
(f)

**Figure:**
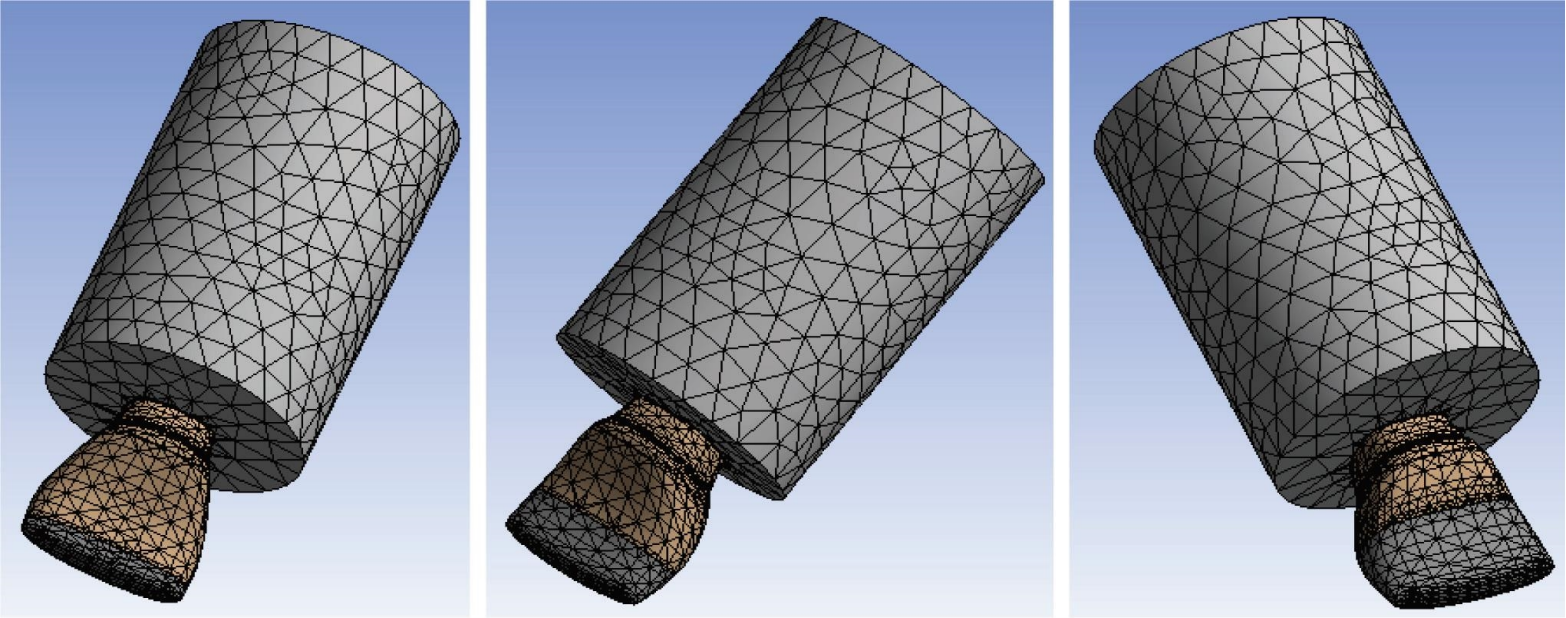
(g)

**Table 2:** Mesh density.

	Model #1 (2 mm)		Model #2 (4 mm)		Model #3 (6 mm)	
Material	Nodes	Elements	Nodes	Elements	Nodes	Elements
Cortical bone	5,391	2,694	5,391	2,694	5,391	2,694
Spongy bone	8,140	4,996	8,140	4,996	8,140	4,996
Root	13,309	12,983	10,681	6,235	9,831	5,731
PDL	6,604	3,381	6,167	3,080	6,167	3,080
Gutta‐percha	223	27	223	27	223	27
Cement (30 *μ*m)	15,027	7,447	11,753	5,786	10,451	5,100
Endocrown	3,507	2,101	2,258	1,148	2,628	1,371

The highest plane of the model (maxillary bone) was considered fixed in the three directions as a boundary condition, while the applied loads were set as 50 N [18], directed with a 135° oblique angle from the vertical plane to the following points:

(1) The lingual slope of the incisal edge
(2) The junction between the incisal and middle thirds

The final models were verified against similar models [7] and showed very good agreement in results. Eighteen linear static analyses were done on a device (Intel Core i7 processor, 2.4 GHz, 6.0 GB RAM) using a commercial multipurpose finite element software package (ANSYS version 16.0).

## 3. Results

A set of 18 calculations was performed for the three models and three materials under two loading cases, where results were extracted and compared to point out the conclusions.

A general notice can be directly withdrawn from results that cortical bone received very high‐level stresses under the 50 N loading. Thus, it is recommended to keep the level of loading less than 20 N for 2 mm height endocrowns and 30 N for longer ones (4 and 6 mm) endocrowns.

**Figure 3:**
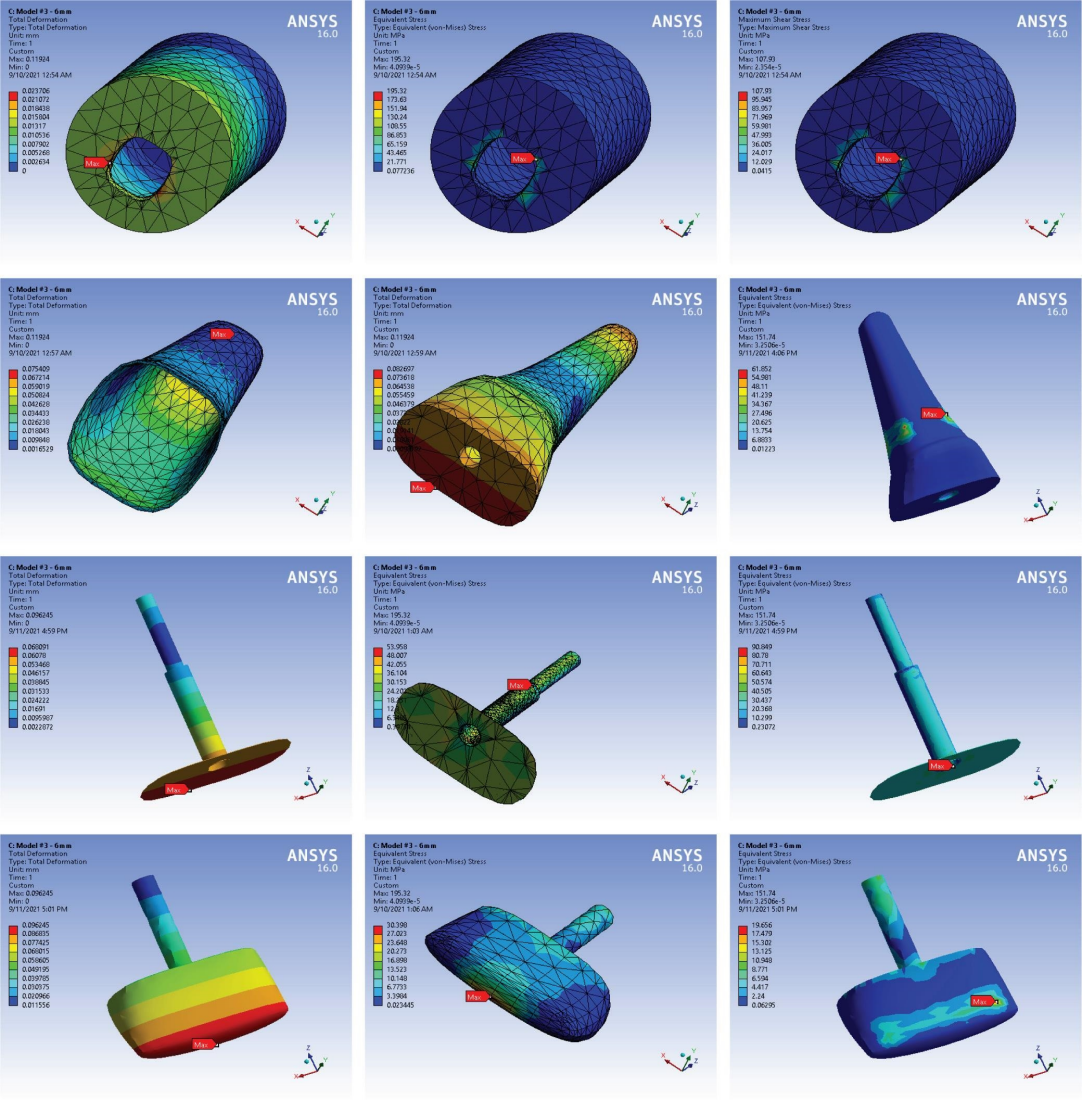
Sample results on models’ components.

The location of extreme values of deformations and stresses on each model component did not change with changing endocrown material or endocrown height, while load location change from tooth tip to junction effect appeared on cement layer and endocrown. The crest of cortical bone and tip of PDL received extreme deformations and stresses. GP ends received maximum stresses at the top and maximum deformation at the bottom. On the other hand, root connection with cortical bone and endocrown received maximum stress and deformation, respectively, as presented in Figure 3.

While the cement layer received the maximum deformation at the connection with the endocrown, the maximum stress moved from the connection at the endocrown tip and the bottom, respectively. Endocrown showed extreme deformation at the tooth tip and maximum stresses under loading points (Figure 3).

**Figure 4:**
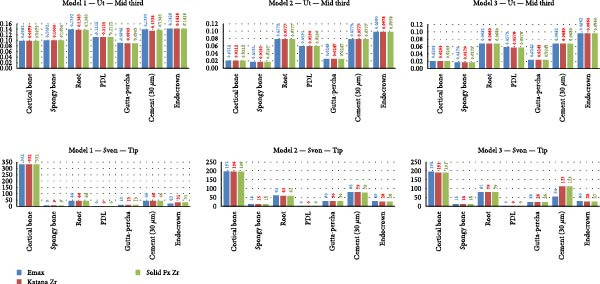
Comparison between the three materials results on all models’ components.

Using different endocrown materials with the same design or height did not show considerable change in total deformation and Von Mises stress, as presented in Figure 4. Minor changes in Von Mises stress on the cement layer and endocrown did not exceed 8% and were recorded with privilege for more rigid (higher modulus of elasticity) material.

**Figure 5:**
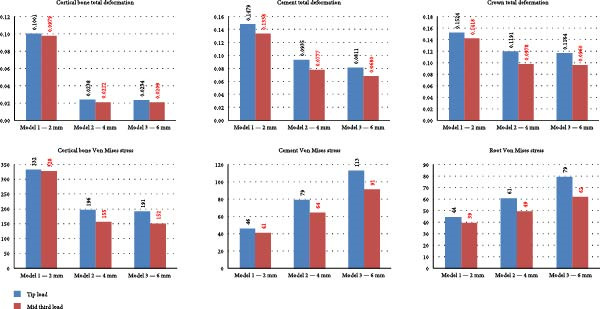
Comparison between the three endocrown height results using Katana Zirconia as indicative results trends.

Values of stresses and deformations vary from one component to the other and from endocrown height to the other but keep general trends. As changing endocrown material has a minor or negligible effect, Katana Zirconia as endocrown material was used in different endocrown heights (comparison in Figure 5).

Total deformation tends to decrease with increasing endocrown height on all the model components, as illustrated in Figure 5. Similarly, Von Mises stress kept the same trend except for root and cement layers.

Deformation differences between Models #2 and #3 were very small and did not exceed 10 *µ*m, while it reached 70 *µ*m between Models #1 and #2. Similarly, the maximum Von Mises stress differences between Models #2 and #3 were negligible (about 2%–5% less on Model #3) except cement layer and root, which reached 45% more on Model #3. In addition, the maximum Von Mises stress differences between Models #1 and #2 were about 30% less on Model #2 except cement layer and root. Model #2 showed more stress by about 60%.

## 4. Discussion

The aim of the research was evaluating the effect of “dental remnant amount” and “restorative material type” on the biomechanics of central incisor restored with endocrown. Using finite element analysis, we could know that the two factors affected the biomechanics of restorations, thereby rejecting the hypotheses in the research. In CAD/CAM systems, we can have prostheses that need less amount of adjusting, better margins, and minute structural defects.

From the machinable blocks, lithium disilicate ceramics with esthetic and mechanical properties stated before clearly in the literature [19]. Recent types of zirconia were assessed being a substitute to lithium disilicate with endocrowns but showed lower adhesion with dentin than lithium disilicate but with higher mechanical strength.

Because of the incisal shape, the retentive features of incisor endocrown restorations must be with more height and narrower compared to posteriors. Related to the endocrowns fabrication method (pressed or machined), the anchorage parts must not be as the metal posts in length, and to be strong, the ceramic must have a larger diameter compared to the post. The need for more teeth structure removal will result in the weakening of the tooth. Therefore, the conventional post and core treatment could still be the best possible option [13].

Our results showed that the different endocrown materials have negligible effects on stress and deformations exerted on the components of the tested models (cortical and spongy bone, PDL, and root). While minor differences were recorded on the endocrown and cement layer that may be referred to as the load transfer mechanism that includes bending and shear. The endocrown material will not affect the bending stress component; it depends only on geometry, while shearing stress slightly affected the transferring media strength. Thus, endocrown material elasticity can play a major role on transferring shear stress to the structures underneath. Crucial stresses in the restoration have been found at the site of the interface between crown‐anchorage elements. This endocrown is liable to be fractured at the cervical site during mastication. These restorations must be fabricated from materials of high strength, like lithium disilicate or recent zirconia [13]. Demachkia et al. [20] opposed this in 2023 where they found that zirconia endocrowns performed better than lithium disilicate endocrowns regarding the fatigue failure load and number of failure cycles. Also, another study opposed this; it was by Taha et al. [21] in 2018, where they found cerasmart resin nanoceramic endocrowns and celtra duo lithium disilicate endocrowns showed better fracture resistance compared to vita Enamic polymer infiltrated endocrowns.

Our results indicate that the location of extreme values of deformations and stresses on each model component changed load location, some of them (on the root, cement, and endocrown) due to changing the load transfer mechanism, as ratios between bending and shear stresses changed. This is in agreement with Zarone et al. [22], who indicated that highly elastic modulus ceramics—zirconium or aluminum oxide must not be anticipating in endocrowns fabrication, as, apart from insufficient bonding to the dentin, they generate crucial concentrated high‐stress at the endocrown–cement–dentin connections.

Regarding the results of this study, as the location of extreme values of deformations and stresses on each model component did not change with changing endocrown material or endocrown height, while load location change from tooth tip to junction effect appeared on cement layer and endocrown, but the Von Mises stress of lithium disilicate transferred to the contact points is less than katana and Zolid Fx due to less modulus of elasticity and more bonding to dentin structures. As a result, contact stresses with the restoration were much less compared to resin cement‐dentin adhesion strength [23, 24].

As regards the amount of remaining dental structure reflected on the height of the endocrown, our results (Figure 3) indicate that the smaller height (2 mm) might be more stable than the larger (4 and 6 mm) one, due to less the stresses on the cement layer. However, the stresses on bone were higher for all materials used. Therefore, the more amount of tooth remnants, the less the stresses concentrated in the cement film [25]. Although the total deformation was not significantly different, it was lower in the lithium disilicate than the Katana and Zolid Fx, due to the lower modulus of elasticity in the former, leading to lower stress transfer to cement type [26].

For sure, bond failure is mainly the cause of failed endocrowns [27]; this feature could be more with Katana and Zolid Fx prostheses, as stresses within the bond interface are a little bit more, so that made lithium disilicate a potential substitute.

Zhu et al. [28] studied the biomechanical behavior of endocrowns with upper premolars, discovering that how thick is the restoration influenced how stresses are distributed. When comparing thicker endocrowns to thinner restorations, the authors found that the thicker endocrowns contained more tension, resulting in lower stress on surrounding tissues, implying no possible future separation.

Clinical research on endocrowns for the restoration of incisors is limited. In terms of biomechanics, a number of researchers believe that endocrowns with anteriors operate as small‐length posts. Fracture resistance of teeth treated using small length posts is two to five times less than that of those restored with conventional length posts that make up two‐thirds of the root length [13, 29]. The advancement of adhesive technologies and higher‐strength ceramics has facilitated the creation of endocrowns, which can now be used as a substitute to traditional restorations, particularly with the posteriors [2]. Another finite element study by Conserva et al. [30] in 2017 showed that the polymerization of composite cores might cause leakage at the margins at the ferrule level that is why endocrowns have an advantage over post and cores.

Using endocrowns with anteriors, however, is yet debatable from the limitation of this study, that it did not include any type of margin preparation. We indicate the need for more research to corroborate the current findings.

## 5. Conclusions

Within the limitations of this research, the following conclusions are shown:

(1) Tooth remnant should be left and conserved.
(2) With thin endocrown, there are concentrated stresses, so bond interface with proper bonding technique needs great care.

## Conflicts of Interest

The authors declare that they have no conflicts of interest.

## Data Availability

The data presented in this study are available on request from the corresponding author. The data are not publicly available due to certain restrictions.
